# Application of Multi-Electrode Resistivity Method on Semi-Diagenesis Rocks in Freezing Area and Its’ Implications

**DOI:** 10.3390/s22145290

**Published:** 2022-07-15

**Authors:** Wenguang Kan, Zailin Yang, Menghan Sun

**Affiliations:** 1 College of Aerospace and Civil Engineering, Harbin Engineering University, Harbin 150001, China; kanwenguang@hrbeu.edu.cn (W.K.); yangzailin00@163.com (Z.Y.); 2 School of Water Conservancy and Civil Engineering, Northeast Agricultural University, Harbin 150030, China

**Keywords:** frozen soil, semi-diagenetic rocks, electrical resistivity, mechanical analysis, bearing capacity

## Abstract

Recently, with the development of geophysical exploration technology, geophysical engineering instruments and methods have also improved. The multi-electrode resistivity method is widely used in engineering exploration. In this paper, multi-electrode resistivity tests were carried out in a seasonal frozen soil area in Heilongjiang Province, to provide an optimized multi-electrode resistivity method under the conditions of frozen soil. Combined with shear wave velocity tests and standard penetration tests, multi-electrode resistivity tests were used to comprehensively analyze and evaluate the physical and mechanical properties of Tertiary semi-diagenesis rocks. The results show that the high resistivity due to the frozen surface layer acting as a shield can be eliminated by technical means. It is feasible to test the resistivity through the frozen surface layer. The multi-electrode resistivity method can visually reflect the interface between saturated sand and semi-diagenetic rocks. Dividing the interface between saturated sand and semi-diagenetic rocks is advantageous as the morphology of the resistivity curve has a significant curvature change. There is a strong correlation between the resistance and shear wave velocity of a strata in which the Pearson correlation coefficient is as high as 0.99. The multi-electrode resistivity method test used in combination with the shear wave velocity test and the standard penetration test could give the bearing capacity and frictional resistance of semi-diagenetic rocks, which saves a lot of time and material costs in engineering exploration.

## 1. Introduction

Semi-diagenesis rock, formed mainly in the Tertiary, is a kind of partly cemented sediment rock that displays distinctive textures, structures, and mechanical properties [1,2,3]. Since the engineering properties of semi-diagenetic rocks are different from that of loose Quaternary sediments or tough rocks, investigations into and the determination of the physical parameters of semi-diagenesis rocks are lacking [1,2,3]. Currently, one of the applicable methods is drilling to collect strata samples, and then, field descriptions, in situ tests, and laboratory experiments would be carried out. Drilling is a common and reliable process, but it is also expensive. Additionally, the transport of drilling machines is very difficult in blocked fields. Moreover, in a cold region, more disadvantage factors, such as cold weather, short days, frozen water, frozen diesel, etc., will consume a lot of time and materials, which is a huge waste of resources.

Recently, with the development of geophysical detecting technology, geophysical instruments and methods for engineering have also seen substantial improvements [4,5]. Especially in the recent decade, the multi-electrode resistivity method has been widely applied in engineering surveys and uses the resistive diversity between rocks to study the distribution characteristics of underground geologic bodies [6,7,8]. In contrast with the conventional resistivity method, the multi-electrode resistivity method could obtain data automatically and quickly, with the electrode layout completed only once [9,10]. Additionally, the data are preprocessed automatically and exported as diagrams available in various formats. Due to the advantages, e.g., low cost, high efficiency, comprehensive information, and convenient explanation, it is widely used to determine the physical and mechanical parameters in an geological engineering investigation [11,12].

This paper takes a preliminary geotechnical investigation of the super-large Songhua Rive bridge of Tieli-Kezuoyouqi expressway as the research object. Detailed tests based on the multi-electrode resistivity method were carried out in winter. Combined with the shear wave velocity test, the standard penetration test, the uniaxial compressive strength test, and drill data, the geological engineering condition and relative parameters were confirmed. This research provides a reference for the exploration of Quaternary and Tertiary semi-diagenesis rocks using the high-density electrical method in cold conditions. Overall, this paper aims to determine the physical and mechanical parameters of Tertiary semi-diagenesis rocks and presents the potential application of the multi-electrode resistivity method in frozen soil areas.

## 2. Methods and Principle

### 2.1. Principle

The resistivity method is an important branch of electrical exploration. The resistivity method recognizes the distribution and occurrence of blocks under the conditions of an artificial excited electric field and thus provides geological and hydrogeological engineering information. The principle of the resistivity method, regardless of the type of device, is to supply power underground using injection electrodes (A, B) and to measure the voltage difference (ΔU_MN_) between measurement electrodes (M, N). Thus, the apparent resistivity ($\rho_{s}^{AB}$) can be calculated and expressed as follows:

(1) $$\rho_{S}^{\mathrm{AB}}=K_{\mathrm{AB}}\;\frac{{\Delta U}_{\mathrm{MN}}}{I}$$

where K_AB_ is the device coefficient and I is the current intensity. When the Wenner electrode array (AM = MN = NB = a) is adopted, K_AB_ is 2πα, in which α represents the distance between adjacent electrodes. Indeed, the multi-electrode resistivity method is a kind of array exploration method that needs to place all electrodes (dozens to hundreds of electrodes) on every measuring point of an observation profile in the field measurements and that collects data fast and automatically using a program-controlled electrode multiplexer and a microcomputer electrical measuring instrument. When the data are fed to the microcomputer, according to the measured apparent resistivity, the resistivity distribution in the strata can be calculated and analyzed so that the strata and abnormal geological blocks can be determined.

In our measuring process, the α array in the fixed section scanning measurement, e.g., an electrode array, was used. A total of 60 electrodes were set at one time with a point distance of 4 m. In contrast with the normal resistivity method, the multi-electrode resistivity method sets a higher density of measuring electrodes, which has higher accuracy and anti-interference ability and can obtain abundant geoelectric information. This method can not only reveal the strata change along the horizontal direction but also reflect the strata change in the vertical direction at certain depths. The data processing using ‘CT’ technology makes resistivity images, which could clearly, intuitively, and accurately reflect the resistivity distribution of geoelectric sections.

### 2.2. Instrument

#### 2.2.1. Multi-Electrode Resistivity Test

**Figure 1:**
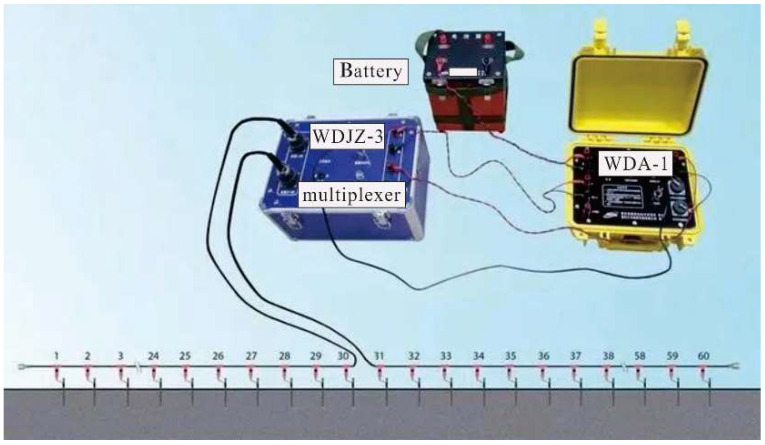
Schematic diagram of measuring system.

**Figure 2:**
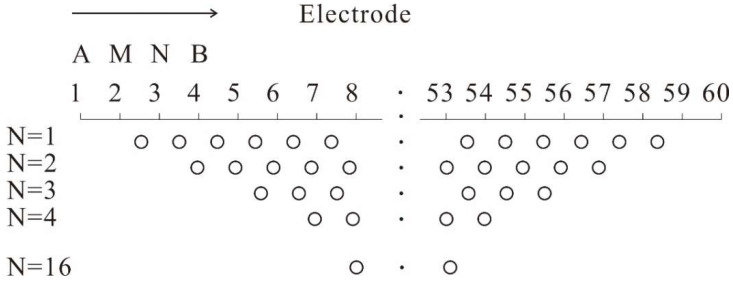
α array for multi-electrode, locations attributed to apparent resistivity measurements.

The test instrument for the multi-electrode resistivity method uses a WDA-1 super digital direct current meter made by Chongqingbenteng Numerical Control Technology Research Institute as the host machine with accessories such as a WDZJ-3 multi-channel electrodes multiplexer, a high voltage DC power supply, a centralized 32 core cable, and copper electrodes (Figure 1). As shown in Figure 2, the host machine assigns A, B, M, and N electrodes through a RS232 multiplexer. Additionally, the multiplexer is connected with electrodes numbered from 1 to 60. If the number of electrodes needs to be expanded, another WDZJ-4 multiplexer can be connected to the multiplexer by connecting the former post RS232 socket to the latter pre RS232 socket using a dedicated cable. At the same time, all of the A, B, M, and N sockets of WDZJ-4 multiplexers are connected with wires correspondingly. The Wda-1 host machine automatically encodes electrodes connected with the new WDZJ-4 multiplexer from 61 to 120.

#### 2.2.2. Standard Penetration Test (SPT)

The standard penetration test (SPT) is a type of dynamic penetration test method in the field that is used to measure the bearing capacity of sand and clay soil foundation. In the test process, it adopts a certain hammering power (hammering weight 63.5 kg and falling distance 76 cm) to hit the strata. When drilling to the certain soil layer required for the standard penetration test, the drill hole was cleaned and then the standard penetration device was use. First, the penetrator hit the test soil 15 cm deep. Then, it began to penetrate 30 cm into the soil and to count the number of hits. The number of hits was recorded and was used as the standard number of hits. The soil sample was taken out of the penetration device to identify and describe the soil.

#### 2.2.3. Shear Wave Velocity Test

The shear wave velocity test is an in situ geophysical exploration method of drilling a hole. We used a RSM-24FD wave velocity tester produced by the Institute of Rock and Soil Mechanics, Chinese Academy of Sciences. Additionally, the probe and sensor were a JBT down hole test probe and a JBC1 sensor produced by Institute of Engineering Mechanics, China Earthquake Administration. During the test, the three-component sensor was placed in the drilling hole closely. A wood strip was placed on the ground 2 m away from the drilling hole and pressured by weights. By hammering both ends of the strip to produce a vibrating wave, the time–history curve of the vibrating wave was recorded by the sensor [13,14].

## 3. Experimental Program

### 3.1. Engineering Information and Geological Condition

The proposed Songhua River Bridge is located about 9 km southwest of Tonghe County, Heilongjiang province, Northeast China, and crosses the Songhuajiang River. The bridge is 4281 m long and 32 m wide, and its structure consists of prestressed concrete simply supported by a continuous box girder. The foundations of the bridge use cast in situ bored pile.

**Figure 3:**
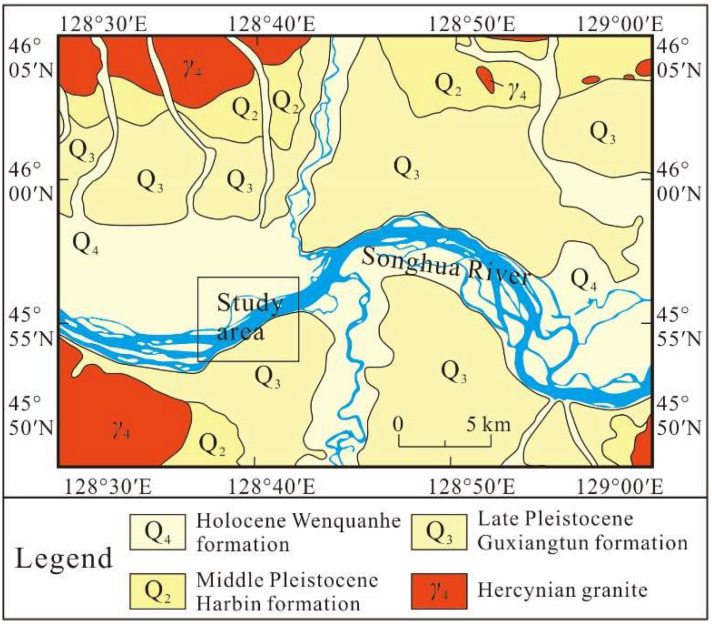
Geological map of the study area.

Tectonically, the study area is at the junction between the Siberian, North China, and Pacific plates. The geographic and geomorphic setting is controlled by the Songhua River (Figure 3). Since the Cretaceous, thousands of meters of sediments have been deposited [15]. The 1920 m thick Early Cretaceous Taoqihe Formation is considered the bottom of the sediments and is composed of siltstones, sandstones, and conglomerates. The Paleocene–Eocene Dalianhe Formation with a thickness of 2000 m overlaid the Taoqihe Formation uncomformably, which is a sequence of mudstones and sandstones, interbedded with coal seams. The Oligocene Dingshancun Formation overlaid the Dalianhe Formation conformably, which is a sequence of pebbly sandstones, medium sandstones, fine sandstones, and mudstones with lignites. The Quaternary strata are widely developed in Heilongjiang Province, which could be divided into the early Pleistocene Dongshenjing Formation, the middle Pleistocene Harbin Formation, the late Pleistocene Guxiangtun Formation, and the Holocene Wenquanhe Formation. The Quaternary strata are mainly composed of clay, silt, fine sand, coarse sand, and pebble.

The Dingshancun Formation is a sequence of semi-diagenesis rocks. Although the rocks have some basic characteristics of bedrock, its mechanical strength is obviously different from that of bedrock. It has low strength, can be broken easily, and has drill rock that is generally columnar or fragmented. The rocks are thick bedded and sandy structures, mainly composed of quartz (80%), feldspar (10%), mica (5%), and cement (5%). With an increase in depth, the semi-diagenesis rocks gradually change to normal sandstones.

The drilling results show that the main semi-diagenetic rocks are sandstones, which are yellow brown or gray and have medium thicknesses to thick bedded, sandy structures and argillaceous and silicic weak cement. The major minerals are quartz, sericite, and kaolinite. After soaking in water, the rock core is spread and could be easily crushed by a hand. The poor diagenesis mudstone layers interbedded with the sandstones are dark gray or black. Additionally, it would have cracks after air drying, similar to hard clay. Mudstones can be softened after soaking in water.

### 3.2. Test Process for Electrical Resistivity

**Figure 4:**
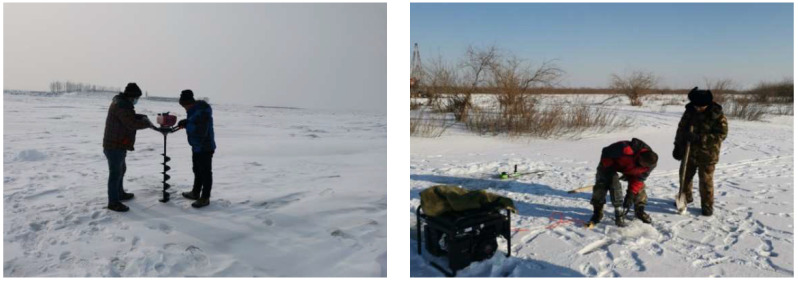

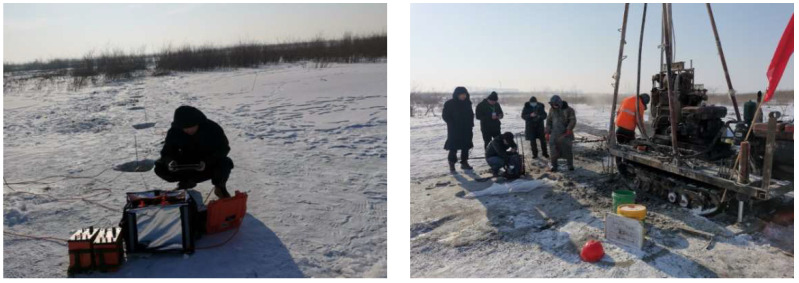
The multi-electrode resistivity method test and shear wave velocity test in the field.

The study area is located in the northern part of Northeast China, where the winter is cold and the daytime temperature is at about −22 °C. The average thickness of the frozen soil layer was about 0.75 m. Geophysical instruments cannot work at conditions under −10 °C. Additionally, the resistivity shielding effect of permafrost also seriously interferes with geophysical data collection, which would yield vortex closed anomalies and would distort measured data. To solve these problems, in the test process, cotton-padded clothes were put on the host and multiplexer. Additionally, a transparent computer insulation box was created and used for a good insulation effect. Moreover, the ice layer was drilled by an ice drill, and the frozen soil layer was drilled by an electric pick. Then, a thick steel pike was inserted into the unfrozen soil, and the electrode was connected to the upper outcrop of the pike. Thus, good high-density electrical data were obtained (Figure 4).

During the test process, a Wenner array with 60 electrodes was used, with a point spacing of approximately 4.0 m. The maximum power voltage and current of the instrument were 900 V and 5 A, respectively. Additionally, the output power was 200 V/200 mA. A RES2DINV automatic iterative inversion program was used for data processing, in which apparent modeling and inversion modeling were based on the finite element analysis method and the smooth constrained least-square inversion method. In addition, the results were constrained using known resistivity ranges of the strata with three iterations. Additionally, the standard penetration tests and shear wave velocity tests were carried out sequentially.

## 4. Results and Discussion

**Figure 5:**
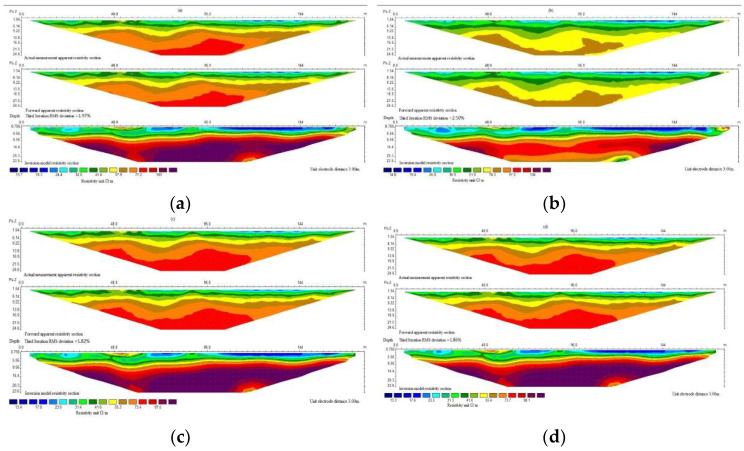
Apparent and inversed diagrams of resistivity of typical sections: (**a**) the apparent and inversed resistivity maps of the typical sections without a frozen soil layer; (**b**) the apparent and inversed resistivity maps across a partially frozen soil layer with a frozen soil thickness of 25 cm; (**c**) the apparent and inversed resistivity maps across a partially frozen soil layer with a frozen soil thickness of 50 cm; (**d**) the apparent and inversed resistivity maps with a 75 cm-deep frozen soil layer.

As shown in Figure 5, the contours of the resistivity image are relatively gentle without abrupt fluctuation, and changes from low- to high-resistance areas are gradual. The layered structure of the terrain resistivity was obtained by inversion, which is consistent with the drill profile. The results show that the resistivity of saturated sand linearly increases with the increase in depth from a small value (from slightly less dense to moderately dense) to a bigger value (for compact sand). At 15 to 20 m depth, which is the top of the semi-diagenesis rocks, the resistivity stops increasing and remain constant. The resistivity of saturated sand increases with the decrease in porosity because the smaller the porosity, the worse the pore connectivity, which reduces the conduction through the pore fluid, resulting in an increase in resistivity of the sand. In the case of semi-diagenetic rocks, the sand grains were completely compacted, so porosity cannot be reduced further. Therefore, their resistivity value stops increasing and is largely equivalent to that of dense sand.

**Figure 6:**
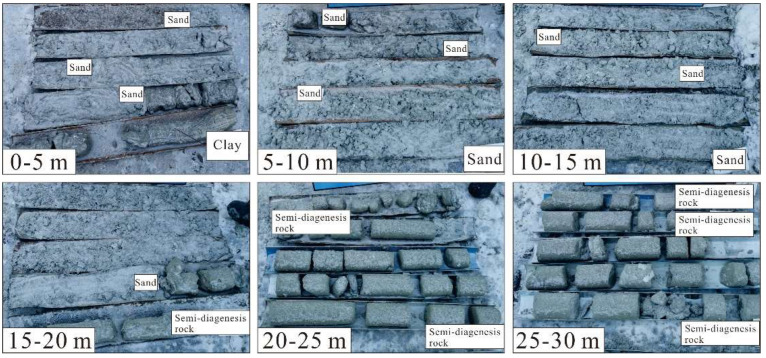
Photos for borehole lithology.

Twenty-one drill holes, 70 to 100 m deep, were made in three rows to identify the strata and to verify the results of resistivity (Figure 6). The uniaxial compressive strength of natural semi-diagenetic rocks is generally less than 3 MPa. The characteristics of the rocks are shown in Table 1.

Shear wave velocity tests from ground to shallow semi-diagenetic rocks were conducted. Standard penetration tests were carried out for sand soil and shallow semi-diagenetic rocks within 25 m under the ground. The resistivity test and borehole test of our geotechnical investigation show that the rocks have poor diagenesis and show partial engineering properties of soil. The results, their relationships, and the engineering property parameters of the sand strata and semi-diagenetic rocks are analyzed and discussed in the following.

### 4.1. Correlation between Resistivity and Shear Wave Velocity

**Figure 7:**
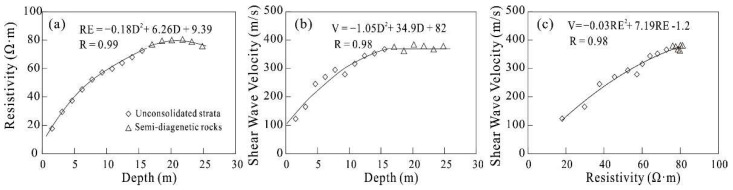
Electrical resistivity versus depth, shear wave velocity versus depth, and electrical resistivity versus shear wave velocity.

The result of the shear wave velocity test is shown in Figure 7. In unconsolidated strata, the value of the shear wave velocity increases with depth. In the top surface of semi-diagenetic rocks, the shear wave velocity does not show obvious increases, which could be used to determine the interface of the rocks. The results indicate that there are correlations among wave velocity, depth, and resistivity.

The resistivity of semi-diagenetic rocks is close to that of dense sandy soil and significantly lower than that of rocks [16]. Meanwhile, for the unconsolidated strata, the results show that there is a strong correlation between resistivity and depth, and the Pearson correlation coefficient (R) is as high as 0.99. The relationship between the two can be expressed as RE = −0.18D^2^ + 6.26D + 9.39, where RE is resistivity and D is depth. It shows that the resistivity of strata is very strictly controlled by depth (Figure 7a). However, for semi-diagenetic rocks, there is a very weak negative correlation between resistivity and depth (R = −0.25).

There is also an obvious positive correlation between shear wave velocity and depth for the unconsolidated strata (R = 0.98), and the relationship between them can be expressed as V = 1.05D^2^ + 34.9D + 82, where V is shear wave velocity and D is depth (Figure 7b). For semi-diagenetic rocks, there is no correlation between shear wave velocity and depth (R = 0.18). Moreover, the correlation between shear wave velocity and resistivity is higher for unconsolidated strata and semi-diagenetic rocks, where V = −0.03RE^2^ + 7.19RE − 1.2 (Figure 7c), suggesting a direct link between these two petro-physic parameters.

### 4.2. Comparative Analysis of Resistivity and Standard Penetration Test

**Figure 8:**
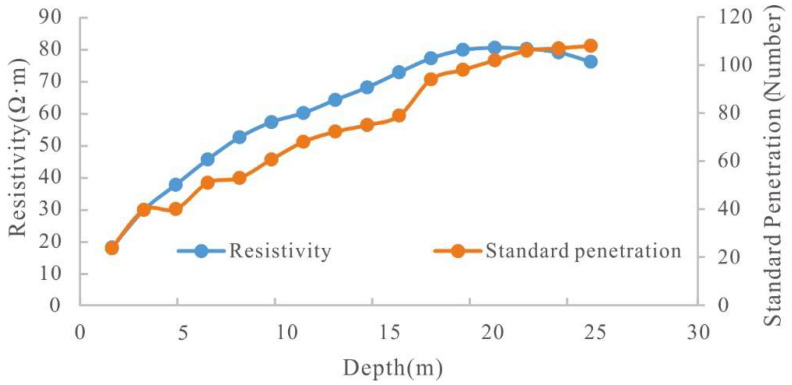
Electrical resistivity versus standard penetration.

The results of the standard penetration test are shown in Figure 8, which illustrates the relationship between resistivity and bearing capacity. As the variation in semi-formed rock density is within a small range, the number of standard penetration test hits slowly increases with depth. Therefore, it can be used to recognize semi-diagenetic rocks. The correlation between resistivity and standard penetration hit number can provide support for judging the rock and soil physical states.

### 4.3. Mechanical Properties of Semi-Diagenetic Rocks

Semi-diagenetic strata have lower diagenetic degrees. They undergo rare tectonic events, which are generally not joints or folds. They have the characteristics of both soil and rocks in terms of composition and structure. Due to the poor rock diagenesis and mechanical engineering properties, the bearing capacity was determined after a comprehensive analysis and an evaluation of the obtained data. The results are shown in Table 1.

## 5. Conclusions

In this study, the application of the multi-electrode resistivity method in frozen soil was optimized, and the geological stratification interface of Tertiary and Quaternary strata was recognized using the resistivity method. Comprehensive analyses and an evaluation of the mechanical properties of Tertiary semi-diagenesis rocks were conducted using multi-electrode resistivity method tests, shear wave velocity tests, and standard penetration tests. The conclusions are listed as follows:

(1) The high resistance generated by surface frost soil disturbs the resistivity of the underlying soil layer. It is feasible to use technical means to penetrate the surface frost layer to test resistivity.

(2) The multi-electrode resistivity method can intuitively reflect the interface between the saturated sandy soil and semi-diagenetic rocks. The interface is shown by the shape of the resistivity curves where a relatively significant change in curvature is observed. The results of the multi-electrode method in the saturated sand layer reflect the positive correlation with shear wave velocity and the number of hits in standard penetration.

(3) The multi-electrode resistivity test, the shear wave velocity test, and the standard penetration test were used to comprehensively analyze and evaluate the bearing capacity and frictional resistance of semi-diagenetic rocks.

## Figures and Tables

**Table 1:** Mechanical properties of semi-diagenetic rocks.

Stratum	Name	State	Characteristic Value of Subsoil Bearing Capacity (kPa)	Standard Value of Pile Lateral Friction Resistance (kPa)
2	fine sand	slightly dense	130	40
2-1	mealy sand	slightly dense	115	25
2-2	silty clay	plastic	205	55
3	medium sand	moderately dense	285	60
3-1	coarse sand	moderately dense	340	80
3-2	fine sand	moderately dense	155	45
4	coarse sand	dense	365	90
5	sandstone (hemidiagenetic)	requires hand to break; fragile	455	115
5-1	mudstone (hemidiagenesis)	requires hand to break; fragile	390	105
6	sandstone (hemidiagenetic)	requires hammer to break; fragile	520	155
6-1	mudstone (hemidiagenesis)	requires hammer to break; fragile	455	130
7	mudstone (hemidiagenesis)	requires hammer to break; fragile	520	155
8	sandstone	requires hammer to break; solid	650	180

## Data Availability

Not applicable.
